# Caregiver Burnout in Pediatric Laryngomalacia: The Role of Clinical and Psychological Factors

**DOI:** 10.7759/cureus.48749

**Published:** 2023-11-13

**Authors:** Yaşar Kemal Duymaz, Ferhat Küçük, Şamil Şahin, Burak Erkmen, Gamze Akçay Oruç, Ayşe Nilüfer Özaydın, Serap Önder, Aslı Şahin Yılmaz

**Affiliations:** 1 Otolaryngology, University of Health Sciences, Umraniye Training and Research Hospital, Istanbul, TUR; 2 Otolaryngology, Private Practice, Istanbul, TUR; 3 Psychiatry, Private Practice, Istanbul, TUR; 4 Preventive Medicine, Marmara University School of Medicine, Istanbul, TUR; 5 Otolaryngology, Acibadem Ataşehir Hospital, Istanbul, TUR; 6 Otolaryngology, University of Health Science, Lütfi Kırdar Training and Research Hospital, Istanbul, TUR

**Keywords:** comorbidity impact, caregiver depression, hamilton depression scale, hamilton anxiety scale, supraglottoplasty treatment, pediatric stridor, chronic health conditions, zarit burden interview, caregiver burnout, laryngomalacia

## Abstract

Introduction: This study aims to assess caregiver burnout in relation to children diagnosed with laryngomalacia and identify factors influencing burnout levels.

Materials and methods: A cohort of 101 caregivers of children with laryngomalacia was studied. Burnout was assessed using the Zarit burden interview (ZBI), while the Hamilton anxiety and Hamilton depression scales were employed to gauge psychological distress. The relationship between burnout and variables like the severity of laryngomalacia, the presence of comorbidities, the child's age, and caregiver demographics was examined using statistical tools in SPSS Statistics version 28.0 (IBM Corp., Armonk, NY, USA).

Results: Caregiver burnout decreased as the child's age increased. A direct correlation was observed between the severity of laryngomalacia and caregiver burnout. The presence of comorbidities in children increased caregiver burnout. Moreover, caregivers with increased levels of depression and anxiety exhibited higher burnout levels. No significant correlation was found between caregiver burnout and socioeconomic status or educational level.

Conclusion: The severity of laryngomalacia, the child's age, the presence of comorbidities, and caregivers' psychological health are significant influencers of caregiver burnout. Healthcare professionals should offer targeted support to caregivers, addressing both their physical and psychological needs.

## Introduction

Laryngomalacia is the most common cause of stridor among newborns, affecting approximately 45% to 75% of infants with congenital stridor [1]. In laryngomalacia, the supraglottic structures collapse into the airway during the inspiratory phase of respiration, leading to an inspiratory stridor [2]. While most infants with laryngomalacia present with mild symptoms and improve between 12 and 24 months, it is essential to recognize that some cases may follow a more severe course [1]. Supraglottoplasty is the primary surgical treatment. Tracheotomy is rarely implemented, especially in cases where surgical intervention fails or in children with multiple medical complications [3].

A chronic health condition, laryngomalacia, can pose significant challenges not only for the child but also for their caregivers. The continuous need for monitoring and care can induce psychological issues in caregivers, such as burnout, anxiety, and depression [4]. This can adversely impact the quality of life for the caregiver, which in turn may reflect on the overall care quality of the child.

Burnout has been defined by Maslach and Jackson (1981) as a three-component syndrome: emotional exhaustion, decreased sense of personal accomplishment, and depersonalization [5]. The Zarit burden interview (ZBI) is a reliable tool frequently used to assess caregiver burnout [6]. Studies have demonstrated that caregivers of individuals with chronic illnesses experience higher rates of burnout [7].

In the literature, there are limited studies assessing the burnout status of caregivers for children with laryngomalacia [4,8]. The primary aim of this study is to delineate the prevalence and degree of burnout among caregivers of children diagnosed with laryngomalacia, seeking to bridge the knowledge void in this domain. Through a nuanced examination of the intricate relationship between caregiver burnout and the clinical severity of the child's condition, along with other potential contributory factors such as comorbidities, socio-economic status, and the caregiver’s mental health, this research endeavors to highlight areas where interventions may be most beneficial.

## Materials and methods

This study was conducted to thoroughly evaluate the burnout status among caregivers of children who have been professionally diagnosed with laryngomalacia. The inclusion of participants was contingent upon several criteria to enhance the validity and reliability of the research outcomes. Caregivers were required to possess a substantial understanding of laryngomalacia, as evidenced by their ability to articulate key aspects of the condition. They also needed to provide informed consent, demonstrating their voluntary commitment to the study and acknowledging their comprehension of the investigative process and their role within it. Active engagement in the care of their child was essential, denoting daily involvement in caregiving responsibilities. Caregivers not meeting these prerequisites, such as those unable to consent, those undergoing psychiatric treatment, or those with limitations preventing full participation, were excluded to preserve the study's integrity.

We established specific exclusion criteria to maintain the study's integrity. Caregivers who either failed to provide informed consent, were actively undergoing psychiatric treatments, or faced other circumstances that prevented them from fully participating in the research were not considered eligible. Before initiating the research, we ensured all ethical considerations were met and obtained the necessary approvals from the appropriate ethical committee (approval no. B.10.1.TKH.4.34.H.GP.0.01/209).

The diagnostic phase was intricate. Two seasoned ENT specialists, each with a minimum of five years of experience with a focus on pediatric airway disorders, were entrusted with diagnosing and classifying the severity of laryngomalacia in the children. Their diagnosis was based on an amalgamation of sources: a detailed medical history of the child, an examination of video recordings that showcased the child's respiratory patterns, and a thorough review of pertinent medical reports and tests.

To gain insight into the caregivers' psychological well-being, we utilized the ZBI score as our primary assessment tool [6]. This tool specifically measures the physical, emotional, and financial burden experienced by caregivers. In addition, to ensure a more holistic understanding of the caregivers' mental health, the Hamilton anxiety and Hamilton depression scales were meticulously administered to each caregiver [9,10]. These assessments were undertaken under the strict supervision or guidance of a psychiatric expert to ensure accuracy.

The participants' Zarit scores then facilitated their categorization into one of three distinct burnout groups, namely mild, moderate, and high. Subsequent analyses were conducted to decipher any potential correlations or patterns. The relationship between the burnout status and various factors such as the severity of the child's laryngomalacia, any concurrent comorbidities, the emotional proximity of the caregiver to the child, the caregiver's educational background, and the family's overall socioeconomic status was keenly examined.

Statistical analysis

For descriptive statistics, we calculated the mean, standard deviation, median, minimum and maximum values, frequency, and percentage to summarize the data. The normality of the distribution of variables was assessed using the Kolmogorov-Smirnov test. To compare quantitative data, we employed the Kruskal-Wallis test and the Mann-Whitney U test, depending on whether two or more groups were being compared, respectively. The chi-square test was utilized to compare qualitative data. In addition to these tests, we also estimated the 95% confidence intervals (CIs) for all the mean values to indicate the precision of our sample estimates. The margin of error was calculated to reflect the range within which the true population parameter will fall. For the inferential statistics, a p-value of less than 0.05 was considered indicative of statistical significance. All statistical analyses were carried out using SPSS software, version 28.0 (IBM Corp., Armonk, NY, USA).

## Results

The cohort of children diagnosed with laryngomalacia comprised 101 individuals, with 40 females and 61 males. Based on the results of the Zarit burnout scale, 47 individuals experienced mild burnout, 32 experienced moderate burnout, and 22 experienced severe burnout. According to the Hamilton depression scale results, 47 individuals showed no depressive symptoms, 26 displayed mild depressive symptoms, 17 had moderate depressive symptoms, and 11 showed severe depressive symptoms. Based on the Hamilton anxiety scale results, 44 individuals exhibited no anxiety symptoms, 27 demonstrated mild anxiety symptoms, 17 had moderate anxiety symptoms, and 13 presented with severe anxiety symptoms. Details regarding the degree of laryngomalacia, accompanying comorbidities, relationship of caregivers, family structure, education, and socioeconomic status are provided in Table 1.

**Table 1:** Demographic and clinical characteristics of children diagnosed with laryngomalacia and their caregivers

Characteristics		Mean±SD	
Age (3.0 to 22.0 years, median = 7.0)		7.1	2.9
Gender		n	%
	Female	40	39.6
	Male	61	60.4
Laryngomalacia	Mild	46	45.5
	Moderate	34	33.7
	Severe	21	20.8
Comorbidity	(-)	85	84.2
	(+)	16	15.8
Relationship	Mother	85	84.2
	Other	16	15.8
Family	Nuclear	81	80.2
	Extended	20	19.8
Caregiver educational status	Elementary	52	51.5
	High school	34	33.7
	University	15	14.9
Socioeconomic level	Low	56	55.4
	Mid	31	30.7
	High	14	13.9
Hamilton depression	(-)	47	46.5
	(+)	54	53.5
	Mild	26	25.7
	Moderate	17	16.8
	Severe	11	10.9
Hamilton anxiety	(-)	44	43.6
	(+)	57	56.4
	Mild	27	26.7
	Moderate	17	16.8
	Severe	13	12.9
Zarit	Mild	47	46.5
	Moderate	32	31.7
	High	22	21.8

In the Zarit mild group, patients' ages were higher than in the Zarit moderate and Zarit high groups (p < 0.05). There was no significant difference in age between the Zarit moderate and Zarit high groups (p > 0.05). The gender distribution across the Zarit mild, Zarit moderate, and Zarit high groups showed no significant difference (p > 0.05). The degree of laryngomalacia in the Zarit mild group was lower than in the Zarit moderate and Zarit high groups (p < 0.05). The degree of laryngomalacia in the Zarit moderate group was lower than in the Zarit high group (p < 0.05). The comorbidity rate in the Zarit mild group was lower than in the Zarit high group (p < 0.05). There was no significant difference in the comorbidity rate in the Zarit moderate group compared to the Zarit mild and Zarit high groups (p > 0.05) (Table 2).

**Table 2:** Comparison of age, gender distribution, degree of laryngomalacia, and comorbidity rates across Zarit burnout groups ^X²^ Chi-square test / K Kruskal-Wallis test (Mann-Whitney U test) ²Difference with Zarit moderate group p < 0.05 ³Difference with Zarit high group p < 0.05

Characteristics		Zarit mild¹		Zarit moderate²		Zarit high³		p	
		Mean±SD		Mean±SD		Mean±SD			
Age		8.0	3.5	6.4	2.1	6.2	1.9	0.018	^K^
		8.0²³		6.0		1.9			
		n	%	n	%	n	%		
Gender									
Female		18	38.3	12	37.5	10	45.5	0.816	^X²^
Male		29	61.7	20	62.5	12	54.5		
Laryngomalacia									
Mild		32	68.1	11	34.4	3	13.6	0.000	^X²^
Moderate		13²³	27.7	15²	46.9	6	27.3		
Severe		2	4.3	6	18.8	13	59.1		
Comorbidity									
(-)		44³	93.6	27	84.4	14	63.6	0.006	^X²^
(+)		3	6.4	5	15.6	8	36.4		

Relationship, family, caretaker educational status, and socioeconomic status distributions across the Zarit mild, Zarit moderate, and Zarit high groups showed no significant difference (p > 0.05). The depression rate in the Zarit mild group was lower than in the Zarit moderate and Zarit high groups (p < 0.05). The anxiety rate in the Zarit mild group was lower than in the Zarit moderate and Zarit high groups (p < 0.05). The depression rate in the Zarit moderate group was lower than in the Zarit high group (p <0.05). The anxiety rate in the Zarit moderate group was lower than in the Zarit high group (p <0.05) (Table 3).

**Table 3:** Comparison of relationship, family structure, caretaker education, socioeconomic status, and mental health indicators across Zarit burnout groups ^ X²^ Chi-square test ²Difference with Zarit moderate group p < 0.05 ³Difference with Zarit high group p < 0.05

Characteristics		Zarit mild¹		Zarit moderate²		Zarit high³		p	
		n	%	n	%	n	%		
Relationship									
Mother		39	83.0	28	87.5	18	81.8	0.816	^X²^
Other		8	17.0	4	12.5	4	18.2		
Family									
Nuclear		38	80.9	26	81.3	17	77.3	0.926	^X²^
Extended		9	19.1	6	18.8	5	22.7		
Caregiver educational status									
Elementary		24	51.1	16	50.0	12	54.5	0.998	^X²^
High school		16	34.0	11	34.4	7	31.8		
University		7	14.9	5	15.6	3	13.6		
Socioeconomic level									
Low		25	53.2	18	56.3	13	59.1	0.929	^X²^
Mid		14	29.8	10	31.3	7	31.8		
High		8	17.0	4	12.5	2	9.1		
Hamilton depression									
(-)		38	80.9	9	28.1	0	0.0	0.000	^X²^
(+)		9²³	19.1	23³	71.9	22	100		
Mild		8	17.0	16	50.0	2	9.1		
Moderate		1	2.1	5	15.6	11	50.0		
Severe		0	0.0	2	6.3	9	40.9		
Hamilton anxiety									
(-)		36	76.6	8	25.0	0	0.0	0.000	^X²^
(+)		11²³	23.4	24³	75.0	22	100		
Mild		9	19.1	17	53.1	1	4.5		
Moderate		2	4.3	4	12.5	11	50.0		
Severe		0	0.0	3	9.4	10	45.5		

## Discussion

This study addresses caregiver burnout and the factors affecting it in children with laryngomalacia. The findings indicate that the demographic and clinical characteristics of both children and caregivers might have varying impacts on burnout levels. As the child gets older, caregiver burnout decreases. Purpura et al., in their study on children with neurodevelopmental disorders, found a negative correlation between the child's age and caregiver burnout [11]. Piran et al., in their study with caregivers of children with chronic illnesses, identified that as the child's age increased, caregiver burnout decreased [12]. Our research indicates that the age of children in the Zarit mild group is significantly higher than that in the Zarit moderate and high groups (p < 0.05). This suggests that caring for older children might be relatively less challenging for caregivers. As children grow older, they become more independent, which may reduce their care needs.

There are contradictory views in the literature regarding the relationship between caregiver burden and the child's gender. Meltzer et al., in their study on adolescents, found that parents of girls with behavioral issues experienced more burnout than parents of boys. On the other hand, they found that parents of girls with emotional issues were less burnt out compared to parents of boys [13]. Esezobor et al., in a study with caregivers of patients with nephrotic syndrome, did not find any impact of the child's gender on burnout. Similarly, our study did not identify a statistically significant difference between both genders [14]. This might be because the children in the study were 24 months old or younger, leading to a lack of gender-specific needs. We speculate that, in adolescents, gender-specific needs might evolve, affecting caregiver approaches.

The severity of the illness increases the caregiver's burden. There is a direct relationship between the severity of laryngomalacia and burnout. Milczuk et al., in their study with caregivers of 44 children diagnosed with laryngomalacia, concluded that as the severity of the disease increased, the quality of life deteriorated [8]. Conway et al. found that, in their study with caregivers of 434 children diagnosed with laryngomalacia, as the disease severity increased, caregivers experienced heightened anxiety and a decline in their quality of life [4]. Similarly, in diseases other than laryngomalacia, such as spina bifida, hemophilia, cancer, and neurological disorders, there's a positive correlation between disease severity and burnout [15-18]. This is understandable, as caring for children with severe symptoms can be physically and emotionally demanding for caregivers. For instance, caring for a child who constantly has breathing issues might require frequent doctor visits, sleep disruptions, or planning special diets in response to feeding difficulties. Health professionals should be aware of this and advise caregivers to seek support.

Comorbidity refers to the presence of one or more additional diseases along with the primary disease. Literature indicates that the presence of comorbid diseases in pediatric patients, including genetic syndromes, asthma, cardiac anomalies, and developmental delays, can significantly increase the burden on caregivers [19-22]. In our study, caregivers of children with comorbidities experienced a higher level of burnout (p < 0.05). The presence of comorbidities in children with chronic diseases can alter the caregiver's daily routine, time management, and even financial burden. This added responsibility can negatively impact the caregiver's mental and physical health. It's not just about extra treatments or doctor visits; managing symptoms caused by comorbidities can also be challenging.

Caregivers can face not only physical but also psychological challenges due to the burden they bear. In particular, the presence of depression and anxiety in caregivers is often indicative of burnout. Many studies in the literature corroborate this observation [23-26]. Our research also found a positive correlation between burnout, depression, and anxiety (p < 0.05). Caregivers, due to the nature of their duties, often find themselves under emotional stress. This stress increases, especially when the care recipient's condition deteriorates or they encounter uncertainties and unexpected challenges in the treatment process. Anxiety, another common condition among caregivers, can be caused by worries about the care recipient, uncertainties about the future, and challenges faced during caregiving. These psychological conditions (depression and anxiety) combined can further increase burnout among caregivers. In conclusion, caregivers' mental health has a significant impact on their levels of burnout. This indicates that support programs for caregivers should focus not only on physical needs but also psychological ones.

The relationship between socioeconomic status, education level, and caregiver burnout is multifaceted and influenced by diverse factors. While a low socioeconomic status is commonly associated with economic hardships, restricted access to healthcare and community resources, and increased social isolation, these stressors can contribute to higher levels of caregiver burnout. Moreover, caregivers from lower socioeconomic backgrounds may encounter additional barriers such as inadequate health literacy and less social support, exacerbating the psychological toll of caregiving [27,28]. Education level serves as another significant determinant of caregiver burden. Higher educational attainment can afford caregivers a better understanding of medical conditions, more effective communication with healthcare providers, and enhanced problem-solving skills, which are essential in managing the complexities of care. Educated caregivers may also possess more robust coping mechanisms and a greater ability to navigate healthcare systems effectively [27,28]. Conversely, higher education can sometimes correlate with increased awareness of caregiving demands and the potential for role strain, particularly when balancing professional responsibilities with care. The expectations and self-imposed pressures to provide high-quality care can also be more pronounced among those with more education, potentially leading to greater burnout [29,30]. In conclusion, socioeconomic and educational factors may potentially influence caregivers' burnout. However, the effect of these factors might vary from one population to another. Finding that these factors don't have a significant impact might be due to specific characteristics of the study population or the research methodology.

This study has some limitations. Firstly, since the data is based on self-reporting, it might be subject to participants' subjective interpretations and recall biases. Also, the lack of ethnic and cultural diversity in our study population might hinder the generalizability of our findings to a broader community. Adopting a cross-sectional design doesn't provide a perspective on how burnout levels evolve over time. Lastly, conducting the study in a limited geographic area or a specific health center might restrict the generalizability of the findings to different geographic or institutional contexts.

## Conclusions

Our study has reveals that as the child's age increases, the caregiver's burnout decreases. The severity of the illness increases the caregiver's burden, and comorbid diseases also have a significant impact on burnout. Additionally, the psychological health of caregivers, especially in terms of depression and anxiety, has been observed to have a significant influence on their burnout levels. It is crucial for healthcare professionals to be aware of the challenges faced by caregivers and to provide them with both physical and psychological support, which can play a critical role in reducing their burnout.

## References

[1] Landry AM, Thompson DM (2012). Laryngomalacia: disease presentation, spectrum, and management. Int J Pediatr.

[2] Thompson DM (2007). Abnormal sensorimotor integrative function of the larynx in congenital laryngomalacia: a new theory of etiology. Laryngoscope.

[3] Jain D, Jain S (2022). Management of stridor in severe laryngomalacia: a review article. Cureus.

[4] Conway RM, White GZ, Thottam PJ (2020). The burden of laryngomalacia and its effects on caregivers: a support group survey evaluation. Int J Pediatr Otorhinolaryngol.

[5] Maslach C, Jackson SE (1981). The measurement of experienced burnout. J Organ Behav.

[6] Rego Filho JF, Sena C, Wajnsztejn R (2023). Burden in caregivers of children with congenital Zika syndrome in Pernambuco, Brazil: analysis and application of the Zarit burden interview scale. PeerJ.

[7] Adelman RD, Tmanova LL, Delgado D, Dion S, Lachs MS (2014). Caregiver burden: a clinical review. JAMA.

[8] Milczuk HA, Johnson SM (2000). Effect on families and caregivers of caring for a child with laryngomalacia. Ann Otol Rhinol Laryngol.

[9] Hamilton M (1959). The assessment of anxiety states by rating. Br J Med Psychol.

[10] Hamilton M (1960). A rating scale for depression. J Neurol Neurosurg Psychiatry.

[11] Purpura G, Tagliabue L, Petri S, Cerroni F, Mazzarini A, Nacinovich R (2021). Caregivers' burden of school-aged children with neurodevelopmental disorders: implications for family-centred care. Brain Sci.

[12] Piran P, Khademi Z, Tayari N, Mansouri N (2017). Caregiving burden of children with chronic diseases. Electron Physician.

[13] Meltzer H, Ford T, Goodman R, Vostanis P (2011). The burden of caring for children with emotional or conduct disorders. Int J Family Med.

[14] Esezobor CI, Solarin AU, Olagunju AT (2020). Significant burden and psychological distress among caregivers of children with nephrotic syndrome: a cross-sectional study. Can J Kidney Health Dis.

[15] Malm-Buatsi E, Aston CE, Ryan J (2015). Mental health and parenting characteristics of caregivers of children with spina bifida. J Pediatr Urol.

[16] Westesson ML, Sparud-Lundin C, Baghaei F, Khair K, von Mackensen S, Mora AM, Wallengren C (2019). Burden on parents of children with haemophilia: the impact of sociodemographic and child's medical condition. J Clin Nurs.

[17] Olagunju AT, Sarimiye FO, Olagunju TO, Habeebu MY, Aina OF (2016). Child's symptom burden and depressive symptoms among caregivers of children with cancers: an argument for early integration of pediatric palliative care. Ann Palliat Med.

[18] Pedrón-Giner C, Calderón C, Martínez-Costa C, Gracia BS, Gómez-López L (2014). Factors predicting distress among parents/caregivers of children with neurological disease and home enteral nutrition. Child Care Health Dev.

[19] Delgado JF, Almenar L, González-Vilchez F (2015). Health-related quality of life, social support, and caregiver burden between six and 120 months after heart transplantation: a Spanish multicenter cross-sectional study. Clin Transplant.

[20] Maltseva M, Schubert-Bast S, Zöllner JP (2023). Sleep quality, anxiety, symptoms of depression, and caregiver burden among those caring for patients with Dravet syndrome: a prospective multicenter study in Germany. Orphanet J Rare Dis.

[21] Datz H, Tumin D, Miller R, Smith TP, Bhalla T, Tobias JD (2019). Pediatric chronic pain and caregiver burden in a national survey. Scand J Pain.

[22] Hewawitharana BD, Wijesinghe CJ, De Silva A, Phillips JP, Hewawitharana GP (2023). Disability and caregiver burden: unique challenges in a developing country. J Pediatr Rehabil Med.

[23] Viny M, Trevino AY, Bouldin ED, Kalvesmaki A, Roghani A, Pugh MJ (2023). Caregiver burden and COVID-19: how epilepsy caregivers experienced the pandemic. Epilepsy Behav.

[24] Matthews A, Bruening AB, Aarnio-Peterson CM, Kramer R (2023). Predictors of caregiver burden before starting family-based treatment for adolescent anorexia nervosa and associations with weight gain during treatment. Eat Weight Disord.

[25] Uzuner S, Durcan G, Sahin S (2021). Caregiver burden and related factors in caregivers of patients with childhood-onset systemic lupus erythematosus. Clin Rheumatol.

[26] Eichholz A, Dudeney J, Jaaniste T (2023). Caregiver psychological burden in pediatric chronic pain: a systematic review and meta-analysis of associations with caregiver sociodemographic and biopsychosocial variables. J Pediatr Psychol.

[27] Basilious A, Villani S, Jang H, Kaberi KM, Malvankar-Mehta MS (2022). Quality of life and caregiver burden in pediatric glaucoma: a systematic review. PLoS One.

[28] Chi DL, McManus BM, Carle AC (2014). Caregiver burden and preventive dental care use for US children with special health care needs: a stratified analysis based on functional limitation. Matern Child Health J.

[29] Pinquart M, Sörensen S (2003). Differences between caregivers and noncaregivers in psychological health and physical health: a meta-analysis. Psychol Aging.

[30] Schulz R, Sherwood PR (2008). Physical and mental health effects of family caregiving. Am J Nurs.

